# The Effect of Prebiotic Supplements on the Gastrointestinal Microbiota and Associated Health Parameters in Pigs

**DOI:** 10.3390/ani13193012

**Published:** 2023-09-25

**Authors:** Dillon P. Kiernan, John V. O’Doherty, Torres Sweeney

**Affiliations:** 1 School of Veterinary Medicine, University College Dublin, Belfield, D04 W6F6 Dublin, Ireland; dillon.kiernan@ucdconnect.ie; 2 School of Agriculture and Food Science, University College Dublin, Belfield, D04 W6F6 Dublin, Ireland; john.vodoherty@ucd.ie

**Keywords:** microbiota, gut health, post-weaning, intestinal dysfunction, pathogen infection, antimicrobial resistance, beneficial bacteria, microbiota metabolites, swine

## Abstract

**Simple Summary:**

The gastrointestinal tract (GIT) is home to a large number of microorganisms, referred to collectively as the GIT microbiota. These microorganisms can be beneficial or potentially harmful to the host. Ensuring a high level of microbial diversity in the GIT, with a high abundance of beneficial and a low abundance of pathogenic microorganisms, is essential for host health. A healthy microbiota is vital at all stages of pig production; however, the post-weaning period is of particular importance. The post-weaning period is a phase during which intestinal dysbiosis can occur, providing an opportunity for harmful microorganisms to colonize and proliferate, leading to poor performance and even mortality. Different microorganisms have different metabolic capabilities, varying in the substrates they break down and the subsequent bioactive metabolites they produce. Therefore, the dietary substrates available to microbes have a significant impact on the microbial composition of the GIT and the subsequent metabolites produced. A prebiotic is a substrate selectively utilized by host microorganisms and conferring a benefit to the host. Prebiotics offer a therapeutic strategy in order to alter the composition of the microbiota, enhancing the proliferation of beneficial microbes and production of host-health-promoting metabolites, which can subsequently limit the proliferation of potentially harmful microbes. There is currently a broad range of different prebiotic classes. These vary in structure and composition and subsequently in the effects exerted on the microbiota. The current review is an overview of the different classes of prebiotics, their potential mode benefits, and the main findings from investigations utilizing them in the pigs’ diets to date.

**Abstract:**

Establishing a balanced and diverse microbiota in the GIT of pigs is crucial for optimizing health and performance throughout the production cycle. The post-weaning period is a critical phase, as it is often associated with dysbiosis, intestinal dysfunction and poor performance. Traditionally, intestinal dysfunctions associated with weaning have been alleviated using antibiotics and/or antimicrobials. However, increasing concerns regarding the prevalence of antimicrobial-resistant bacteria has prompted an industry-wide drive towards identifying natural sustainable dietary alternatives. Modulating the microbiota through dietary intervention can improve animal health by increasing the production of health-promoting metabolites associated with the improved microbiota, while limiting the establishment and proliferation of pathogenic bacteria. Prebiotics are a class of bioactive compounds that resist digestion by gastrointestinal enzymes, but which can still be utilized by beneficial microbes within the GIT. Prebiotics are a substrate for these beneficial microbes and therefore enhance their proliferation and abundance, leading to the increased production of health-promoting metabolites and suppression of pathogenic proliferation in the GIT. There are a vast range of prebiotics, including carbohydrates such as non-digestible oligosaccharides, beta-glucans, resistant starch, and inulin. Furthermore, the definition of a prebiotic has recently expanded to include novel prebiotics such as peptides and amino acids. A novel class of -biotics, referred to as “stimbiotics”, was recently suggested. This bioactive group has microbiota-modulating capabilities and promotes increases in short-chain fatty acid (SCFA) production in a disproportionally greater manner than if they were merely substrates for bacterial fermentation. The aim of this review is to characterize the different prebiotics, detail the current understating of stimbiotics, and outline how supplementation to pigs at different stages of development and production can potentially modulate the GIT microbiota and subsequently improve the health and performance of animals.

## 1. Introduction

The gastrointestinal tract (GIT) is home to a complex ecosystem of microbes, including bacteria, archaea, fungi, and viruses, with the GIT microbiota referring to the collection of all these microorganisms [1,2,3,4]. Diversity in the composition of the GIT microbiota is essential for host health, and correlates with a number of extrinsic factors, including diet, age, and body weight [4,5]. The GIT microbiota has an established fundamental role in many aspects of animal production, including feed efficiency [6], growth performance [4] and health status [7]. Establishing a healthy GIT microbiota, that is diverse, with a high abundance of beneficial bacteria and a low abundance of potentially pathogenic bacteria, is a fundamental focus in terms of improving pig health and performance, particularly in the context of reducing antibiotic and antimicrobial use [4,8,9]. It is important to understand not only how and when the GIT is colonized but also the factors that modulate its composition. Current research priorities include: (1) the identification of effective bioactive or bioactive combinations; and (2) the identification of the most effective supplementation period, with the overall objective of establishing and maintaining a healthy microbiota in the pig.

Historically, it was believed that the prenatal pig’s GIT was a sterile environment; however, recent research has suggested that amniotic fluid may offer a small contribution to the colonization of the intestine before birth [10,11,12]. In the immediate postnatal period, the sow’s milk, the sow’s nipples, and the ground environment are the most likely early sources of microbes. However, throughout lactation, the piglet acquires a GIT ecosystem that largely maps to that of their mothers rather than to the housing environment [13]. The sow’s colostrum and milk influence the development of the GIT microbiota through its microbial, nutrient, and prebiotic oligosaccharide composition [13,14]. The suckling pig’s microbiota is dominated by members of the *Bacteroidaceae*, *Clostridiaceae*, *Lachnospiraceae*, *Lactobacillaceae* and *Enterobacteriaceae* genera [15]. 

Weaning occurs at approximately three to four weeks of age on commercial farms. Weaning is abrupt and characterized by dietary, environmental and social changes, all of which can place immense stress on the pig, leading to a disruption of the GIT microbial ecosystem [16]. The switch from a liquid-based milk diet to a typically solid-based plant diet leads to a significant alteration in the substrates available to microbes in the GIT, having a significant impact on the microbial ecosystem [15]. During this period, the microbiota must quickly adapt from a milk-oriented microbiota to a plant-oriented microbiota. This adaptation, combined with the other stressors, provides an opportunity for pathogens to colonize and proliferate, resulting in episodes of diarrhea and even mortality [17,18]. Interestingly, the relative abundance of particular bacterial genera in the suckling pigs’ microbiota, such as *Lactobacillaceae*, *Ruminococcaceae*, *Lachnospiraceae* and *Prevotellaceae*, is associated with a reduced incidence of diarrhea in the pig post-weaning [19]. This suggests that the susceptibility of the suckling pig to pathogenic infection in the post-weaning phase can be alleviated in part by promoting an environment with an increased abundance of these beneficial bacterial genera. The importance of the suckling pig’s microbiota, combined with the limited feed intake in the immediate days post-weaning, underly pre-weaning microbial modulation as a viable strategy for managing the GIT dysbiosis associated with the immediate post-weaning phase. Interestingly, the sow’s microbiota is the predominant contributor to the establishment of the offspring’s microbiota [13], suggesting that modulation of sow microbiota is an effective route for improving the establishment of the offspring’s microbiota [20]. Additionally, enhancing the sow’s microbiota can have multiple health benefits to the sow, thereby enhancing sow performance, prompting further improvements in offspring development, as reviewed in [21].

While the importance of establishing a healthy microbiota is clear, the focus must now be placed on identifying the most effective mechanisms to achieve this goal. Different classes of bioactives with the potential to modulate the microbiota include, but are not limited to, prebiotics, probiotics, synbiotics and stimbiotics [22,23,24,25]. Prebiotics are dietary substrates that are utilized by beneficial microorganisms in the GIT and thereby enhance host health [26]. A probiotic is a live beneficial microorganism which, when administered in adequate amounts, confers a health benefit on the host [27]. Synbiotics are defined as “a mixture comprising live microorganisms and substrate(s) selectively utilized by host microorganisms that confers a health benefit on the host” [28]. Synbiotics are proposed in order to enhance the colonization and survival of the probiotic by providing a prebiotic substrate that can be utilized by the probiotic bacteria and other beneficial microbes [29]. Stimbiotics are a more novel biotic class. They are suggested to act by stimulating fiber-fermenting bacteria to increase their activity and thereby promote fiber fermentation in the GIT [23].

**Figure 1:**
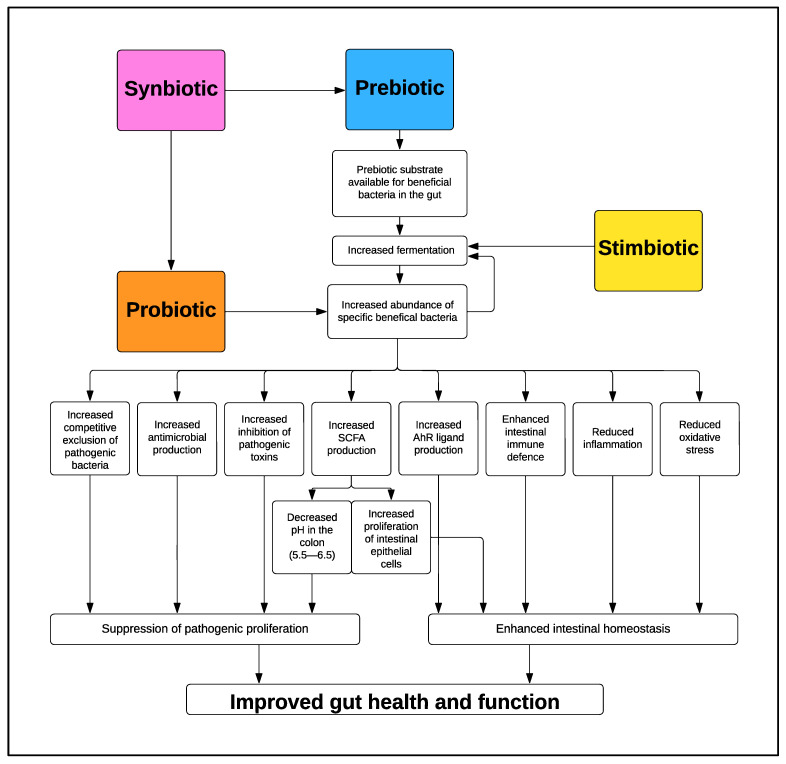
Mode of action of probiotics, prebiotics, synbiotics and stimbiotics in the GIT [21].

Each of these classes of bioactives can potentially increase the abundance of beneficial bacteria and simultaneously decrease the abundance of pathogenic bacteria [24,25,30,31]. While the overall effect of each of these bioactives remains the same, their mechanism of action varies (Figure 1). Modulation of the GIT microbiota is assessed for research purposes by analyzing the microbial composition of the GIT microbiota, quantifying the abundance of bacterial groups, and evaluating microbial diversity [20,32,33,34]. Typically, the concentration of bacterial metabolites in the digesta or feces is also analyzed [20,32,33,34]. The concentration of short-chain fatty acids (SCFAs) is often used as an indicator of the level of fermentation occurring in the GIT and positively correlates with fiber substrate concentrations and/or beneficial fermentative bacteria in the GIT [35]. The aims of this review are to discuss in detail the different types of prebiotics, describe a novel class of microbiota-modulating bioactives known as stimbiotics, and detail how supplementation to pigs at different stages of development can potentially modulate the GIT microbiota and subsequently improve the health and performance of the animal.

## 2. Prebiotics

A prebiotic has traditionally been defined as “a selectively fermented ingredient that results in specific changes in the composition and/or activity of the gastrointestinal microbiota, thus conferring benefit(s) upon host health” [22]. In 2017, an updated definition of a prebiotic was proposed as “a substrate that is selectively utilized by host microorganisms conferring a benefit to the host” [26]. This updated definition expands the categorization of prebiotics from traditionally including only non-digestible carbohydrates to the inclusion of novel prebiotics such as amino acids, peptides, as reviewed in [36], and nucleotides [37]. There is also a case for the inclusion of polyphenols as prebiotics, as reviewed in [38]. Regardless of the preferred definition of a prebiotic, the important aspect of a prebiotic is that it resists digestion in the proximal GIT and can be utilized by beneficial microbes in the distal GIT, enhancing their proliferation and abundance, thereby improving the health of the host. In this review, prebiotics will be divided into traditional and novel prebiotics, with the latter consisting of bioactives that only recently fell under the “prebiotic” classification. 

### 2.1. Traditional Prebiotics 

Traditional prebiotics comprise carbohydrates that are predominantly resistant to digestion by mammalian enzymes [39]. It was originally thought that these prebiotics were completely resistant to mammalian enzymes and reached the distal GIT intact; however, recent studies suggest there may be a degree of degradation of certain traditional prebiotics by brush border enzymes in the small intestine [39,40]. Nonetheless, traditional prebiotics (beta-glucans, non-digestible oligosaccharides, inulin, pectin, and resistant starch) are particularly sensitive to degradation by bacteria in GIT, where they undergo fermentation, leading to the production of host-health-promoting by-products or metabolites [41]. Through the fermentation of the prebiotic, beneficial bacteria obtain energy, which promotes their survival. Through the use of this mechanism, prebiotics selectively influence the composition of the GIT microbiota [24]. These bacteria are beneficial to the host as, via the fermentation of the prebiotic substrate, they can produce health-promoting compounds including SCFA, such as acetate, propionate and butyrate, as well as organic acids such as lactate, succinate and pyruvate. These compounds exert multiple beneficial effects on the host energy metabolism [42,43,44,45]. Although there are many health benefits associated with prebiotic supplementation, the satiety effect of prebiotic fibers must also be taken into consideration when choosing an appropriate inclusion rate, as high inclusion rates may result in a reduction in feed intake and subsequent performance [46,47]. Each traditional prebiotic group exhibits distinct physical and chemical structural characteristics. In addition, there can be physical and chemical structural variations between two similar prebiotics due to differences in the source, extraction protocol and/or production procedure. Structural and chemical properties are crucial in relation to their bioactivity and effect on the GIT microbiota [30,48,49]. It is worth mentioning that some of these bioactives have additional properties, such as antioxidant and anti-inflammatory properties [50,51,52,53,54]. However, for the purpose of this review, the primary focus will be placed on their prebiotic properties.

#### 2.1.1. Beta Glucans (β-Glucans)

Beta-glucans are naturally occurring polysaccharides of D-glucose monomers linked through β-glycosidic bonds. β-glucans are cell wall components of yeast, algae, bacteria, mushrooms, and cereals such as barley and oats [30,55]. β-glucans display a wide range of health-promoting properties, such as anti-inflammatory, antioxidant and prebiotic properties [30,50]. The sugar component of β-glucans is predominantly pure glucose, except for in the case of laminarin, which also contains trace amounts of mannose [56]. The characteristics of the different β-glucans, such as purity, linkage type, degree of branching, structure, solubility, and molecular weight, significantly impact their bioactivities [30,57]. With different forms of β-glucans present in various sources, it is important to isolate the potential benefits of each, rather than grouping β-glucans under a single classification with collective properties. For example, the bonds found in bacteria are predominantly β(1–3) linkages, cereal β-glucans are predominantly β(1–3) and β(1–4) linkages, while in yeast, laminarin and mushrooms, the β-glucans bonds are β(1–3), with β(1–6) branches. Although yeast and laminarin consist of the same type of linkages, the ratio of bonds and branches and the structure of the β-glucans differs [58]. Yeast β-glucans can also be utilized as potential encapsulating agents that can protect another bioactive from digestion, thereby increasing its bioavailability within GIT [59,60]. β-glucan supplementation can improve pig performance by enhancing gut microbial composition [30,61], improving gut morphology and barrier function [33], and also improving immune status [50] in pigs. The biological effects of the supplementation of β-glucans in weaned pigs is an area that has been extensively researched in recent years; however, the maternal supplementation of β-glucans and its effects on offspring is less well documented and is an area that warrants further research. The effects of β-glucan inclusion in the diet of sows and pigs at different stages are summarized in Table 1.

#### 2.1.2. Non-Digestible Oligosaccharides

Non-digestible oligosaccharides (NDO), or functional oligosaccharides, make up a large proportion of the bioactives currently classed as prebiotics. The NDO are a group of oligosaccharides, typically 2–20 monomers in length, with β-links present among the units of monosaccharides. The NDO are distinguished by their monosaccharide composition, chain length, degree of branching, and purity. The NDO can be extracted directly from natural sources or produced via polysaccharide hydrolysis or enzyme processing [78]. For example, xylo-oligosaccharide (XOS) and fructo-oligosaccharide (FOS) are obtained through the enzymatic degradation of xylan and inulin, respectively [79,80]. Non-digestible oligosaccharides have both indirectly and directly beneficial effects on the host’s health. They indirectly benefit the host’s health by acting as a substrate for beneficial bacteria such as *Bifidobacteria* and *Lactobacilli*, thereby promoting their growth and enhancing the health benefits associated with these bacteria [81]. In addition, more direct effects involve reducing the binding sites available to pathogenic bacteria and also direct immunomodulation through binding to receptors that regulate cytokine production [82] and T-cell response [83]. It is suggested that NDO can act as anti-adhesives, preventing the adhesion of certain pathogens to the cell wall in the GIT [84]. Certain NDO are proposed to act as soluble decoy receptors that bind to pathogen receptors and prevent binding to the epithelial layer. Alternatively, it has been suggested that NDO themselves can bind to the epithelial surface and cause structural changes to the receptor, thereby preventing pathogen adhesion [84]. Although these studies provide evidence for direct immunomodulation by NDO, changes in immune markers driven by dietary supplementation are likely due to a combination of both direct and indirect effects. Changes in the GIT microbiota also contribute to changes in immune cell markers [85], and an increase in the abundance of beneficial bacteria leads to increased competition for binding sites, thereby reducing the binding of pathogenic bacteria [86]. There is a wide range of NDO available on the current market, and a number of these have been researched in terms of their effects when included in sow and pig diets in recent years (Table 2).

#### 2.1.3. Inulin 

Inulin is a naturally occurring non-digestible carbohydrate that belongs to the class of dietary fibers known as fructans [101]. Inulin is a polymer that contains both oligosaccharides and polysaccharides. It is a type of fructan mixture that can be found in a wide variety of plants. However, in its industrial use, it is most commonly extracted from chicory roots [102]. Inulin is generally a linear chain comprising one terminal glucose molecule and a chain of fructose units linked by β(2–1) bonds [101]. Inulin’s fructan composition and the number of monomer units, referred to as the degree of polymerization, varies depending on the source [103,104]. The degree of polymerization of inulin can range from approximately 2 to 60 [105]. The FOS is obtained via the enzymatic hydrolysis of inulin, reducing the degree of polymerization [80,106]. The degree of polymerization has a direct influence on the physical properties of the compound. The higher the degree of polymerization of inulin is, the greater its gel-like behavior will be, with longer chains having lower solubility. For this reason, FOS is much more soluble than inulin; FOS is up to 85% soluble at room temperature, while inulin is almost insoluble at room temperature [107,108]. 

When included in sow diets, inulin increases litter performance and improves the antioxidant status of the sow [109]. Inulin has been utilized in weaned and grower pig diets to varying degrees of success [110,111,112,113]. At an inclusion rate of 4%, inulin increases *Lactobacilli* and *Bifidobacteria*, and reduces the presence of harmful *Clostridium* spp. and members of *Enterobacteriaceae* in the intestinal microbiota of grower pigs [111,112]. However, at an inclusion rate of 3%, inulin does not alter the number of *Lactobacilli*, *Bifidobacteria*, *Enterococci*, *Enterobacteria* or bacteria of the *Clostridium Coccoides*/*Eubacterium rectale-group* in the duodenum, jejunum or caecum [113]. The use of short-chain inulin, long-chain and a 50:50 mixture of both all exerted similar effects on the GIT microbiota of pigs in the post-weaning/grower phase, increasing the total number of *Lactobacilli* and *Bifidobacteria,* particularly in the mucosa-associated microbiota [112]. Interestingly, the short-chain inulin influenced the microbiota more proximal in the GIT than the long-chain inulin [112]. Although several studies have observed positive results with the inclusion of inulin in the pigs’ diet, research remains relatively sparse. Further research is warranted, particularly in terms of determining the optimal inulin inclusion rates. The effects of inulin inclusion in the diet of sows and pigs at different stages are summarized in Table 3.

#### 2.1.4. Resistant Starch

Resistant starch is a non-digestible carbohydrate defined as the fraction of starch that resists digestion in the stomach and small intestine and acts as a substrate for bacterial fermentation [117]. There are four types of resistant starch: resistant starch type 1 (RS1) which is found in grains and cereals; RS2, which is found in starch foods, such as banana and potato; RS3, which are retrograded starches that occur when cooking and cooling starchy foods; and RS4, which are man-made chemical resistant starches [118]. A meta-analysis including results from 24 published studies involving RS2 concluded that there is a negative relationship between the RS2 inclusion rate and pH in the large intestine and that increasing RS2 levels promotes fecal *Lactobacilli* and *Bifidobacteria* in pigs [119]. The optimal inclusion rate to achieve these results is suggested to be 10–15% [119]. However, this meta-analysis included studies of pigs covering a broad range of start weights (4.6–105 kg). Resistant starch may be particularly effective in the post-weaning phase, inclusion rates of 0.5–14% raw potato starch improves post-weaning fecal scores [120,121,122]. The microbiota was not analyzed in the study utilizing an inclusion rate of 0.5% [121]; however, an inclusion rate of 5% increases the presence of *Clostridia* in feces [120], and rates of 7 and 14% increase *Lactobacilli* and *Bacteroides* prevalence in the colon [122]. The meta-analysis in [119] is a useful initial indicator of the potential for RS2 supplementation and provides a broad indication of the optimal inclusion rate. Given the broad range of resistant starch sources, further research is warranted to give a more precise indication of what the most effective type and inclusion rate is at different stages of development. The effects of resistant starch inclusion in the diet of sows and pigs at different stages are summarized in Table 4.

#### 2.1.5. Pectin 

Pectin is a plant cell wall polysaccharide that can be utilized by bacteria in the GIT, but which is indigestible to mammalian digestive enzymes. It is present in the cell wall of fruits, vegetables, and legumes [127]. Pectin is a large component of the dietary fiber fraction of feedstuffs such as beet pulp, citrus pulp, and soybean hulls. Citrus and apples are common sources of pectin for use in pig diets [128,129,130,131,132]. The molecular structure of pectin varies depending on its source. The three major pectin structures are homo-polygalacturonate, rhamnogalacturonan I (RGI) and rhamnogalacturonan II [133]. The degree of methyl esterification, the composition of neutral sugars, the degree of branching, and the presence of amide groups all influence the effects of pectin on the microbiota [134]. The cumulative production of the total SCFA and propionate is largest in fermentations of pectin with high methoxyl [134]. The influence of the wide-ranging structural variations present in pectin are reflected in its effects in vivo in terms of variability of findings. Further investigation is required to identify a more precise structure-to-function relationship of pectin supplementation in pigs. The review in [135] provides a detailed summary of results from in vivo and in vitro studies investigating the effects of pectin supplementation on pig GIT microbiota and other health parameters. The potential benefits of the inclusion of pectin in the diet of pigs to the composition of the microbiota is evident [135]; however, the most effective pectin source/structure is less clear. Further research is warranted in order to advance the current understanding of the structure-to-function relationship of pectin and evaluate the most appropriate source for inclusion in pig diets. There have been a number of in vivo pectin supplementation studies in pigs published since the review in [135], which are summarized in Table 5. 

### 2.2. Novel Prebiotics 

Novel prebiotics differ from traditional prebiotics in that they are not non-digestible carbohydrates but are still selectively utilized by host microbes, which can lead to host health benefits. They currently include compounds such as proteins, hydrolysates, peptides, amino acids [60,136,137,138] and nucleotides [37]. Polyphenols have recently been proposed as potentially prebiotic, although, as polyphenols are not currently understood to be utilized by bacteria directly, they are described as having ‘prebiotic-like properties’ [38]. Further research into the mechanism of action of polyphenols and their utilization by microbiota is required. However, for the purpose of this review, polyphenols have been included under the novel prebiotic title. 

#### 2.2.1. Proteins, Hydrolysates, Peptides, and Amino Acids 

The interaction between proteins and the GIT microbiota has been intensely investigated in recent years; although certain modes of action have been suggested, the exact mechanisms remain unclear. Generally, protein digestion and absorption occur in the small intestine, leaving small fractions of protein to transit into the large intestine. Hence, there is a scarcity of amino acids available to bacteria in the distal GIT and competition exists for residual peptides and amino acids among different bacterial groups. This scarcity limits the growth of bacteria and different strains can have specific amino acid requirements [139,140,141]. Recently, the reduction in crude protein levels in the diet of pigs in the post-weaning period has been an area of major research focus [142]. The objective is to reduce the quantity of undigested dietary protein and excess endogenous nitrogen that arrives in the large intestine and is fermented by potentially pathogenic nitrogen utilizing bacteria, thereby reducing their proliferation and the production of toxic metabolites [143,144]. However, reducing dietary crude protein also reduces the amino acid availability for the beneficial GIT bacteria that utilize amino acids to proliferate and produce host-health-prompting metabolites [145]. For example, certain *Bifidobacterium* strains require cysteine for growth [139], while certain *Lactobacillus* strains require a large number of amino acids, particularly arginine, lysine and glutamic acid [140,141]. Very low-protein diets can result in an increase in potentially pathogenic bacteria in the colon, while supplementation with certain amino acids to these low-protein diets, such as valine and isoleucine, above the current recommended levels can help to limit these negative observations [146]. In this regard, a reduction in dietary crude protein should be combined with a specific targeted supply of amino acids to ensure the promotion and maintenance of a healthy microbiota. The potential prebiotic effect of amino acid supplementation is discussed in detail in [36], in which the authors introduce the term “Aminobiotics”.

The extent of hydrolysis and absorption of ingested proteins and amino acids in the GIT prior to reaching the large intestine means that the supplementation of protein or amino acids for the purpose of promoting the growth of beneficial bacteria in the colon is far from optimal, as only small fractions of the supplemented protein or amino acid will be available in the large intestine. However, new techniques for shielding these peptides and amino acids from degradation and absorption have been developed. An example of this is the use of a prebiotic galacto-oligosaccharide (GOS), conjugated with a protein, lactoferrin hydrolysate, that has been pre-hydrolyzed by pepsin [138]. In an aqueous solution, these combinations are suggested to form helical structures, with the GOS component acting as the outer layer with the protein components stored within [147]. This particle structure is suggested to protect the protein from digestive enzymes in the stomach and small intestine, making it indigestible and unabsorbable. The particles are then subjected to digestion by bacteria in the large intestine as the outer layer undergoes fermentation, thereby releasing the inner protein component and making it available to the bacteria [138,147]. The pre-digestion with pepsin reduces the number of pepsin-cleavable bonds and so increases the resistance of the particles to the digestion [138]. The conjugation step, combining lactoferrin hydrolysate and GOS, is suggested to be a key part of the process as an unconjugated combination displayed a 50% slower proliferation of *Lactobacillus casei* compared to the conjugated combination [138].

Although the conjugation was suggested to be a key step in the success of the study in [138], other studies have had positive results when casein hydrolysates are simply supplemented in combination with yeast β-glucan in both sows [136,137] and weaned pigs [60,137]. Interestingly, when supplemented alone, these bioactives have minimal effect, suggesting that a form of natural encapsulation occurs when supplemented together, allowing the yeast β-glucan to act as bioactive carrier for the casein hydrolysate [60]. Maternal supplementation with the bioactive combination of the β-glucan and casein hydrolysate increases the abundance of the phylum *Firmicutes*, including *Lactobacillus* and *Christensella*, in the sow feces, while increasing cecal and colonic abundance of *Lactobacillus* and cecal abundance of *Christensella* in the offspring at weaning time [136]. Maternal β-glucan and casein hydrolysate supplementation also increases the abundance of *Lactobacillus*, decreases the abundance of *Enterobacteriaceae* and *Campylobacteraceae*, and increases butyrate production in the offspring 10 days post-weaning [137]. The casein hydrolysate used in these studies has an established anti-inflammatory effect [148].

The amino acid composition of the casein hydrolysate may play a part in the beneficial effects seen with its supplementation. Casein hydrolysate contains a wide range of different amino acids; the profile varies depending on the degree of hydrolysis and enzymes used [149]. For example, in [150], glutamate and glutamic acid (21%), proline (10.2%), leucine (8.7%) and lysine (7.3%) contribute to 47.2% of the amino acid mass of the casein hydrolysate utilized. The role of amino acids in the diet stretches beyond their function as protein building blocks. They act as energy substrates and signaling molecules and can be metabolized into biologically active compounds, which can promote GIT health [151]. In vitro, branched-chain amino acids (BCAAs: leucine, isoleucine, valine), glutamine, glutamate, and arginine are utilized by microbes originating from the mid-colonic content of grower pigs, resulting in the production of metabolites such as SCFA, further highlighting the potential benefits of amino acid utilization by the microbiota [145].

Tryptophan is an amino acid that has received increased attention over the past number of years due to the beneficial effects of the metabolites produced via the bacterial tryptophan metabolism in the GIT [152,153,154,155]. Tryptophan metabolism by the GIT microbiota is a source of aryl hydrocarbon receptor (AhR) ligands, with AhR being recognized as having important roles in the regulation of intestinal homeostasis, as reviewed in [156]. Microbiota-derived AhR ligands are typically indole derivatives, such as indole-3 ethanol (IE), indole-3 pyruvate [157], indole-3 aldehyde (I3A) and tryptamine (TA) [158]. These ligands can stimulate the AhR, leading to enhanced intestinal barrier function [159,160] and reduced inflammation [161]. However, ensuring the appropriate level of AhR stimulation is important, as overstimulation can potentially lead to intestinal dysregulation [162].

The potential benefits of enhancing the abundance of tryptophan-metabolizing bacteria in the GIT microbiota is a promising strategy with which to stimulate the AhR and promote intestinal homeostasis. Increasing tryptophan content in weaned pig diets has been shown to improve average daily feed intake (ADFI) and average daily gain (ADG) [152]. In the cecum and colon, tryptophan supplementation enhances alpha (α) diversity, increases *Prevotella, Roseburia*, and *Succinivibrio* genera, reduces *Clostridium sensu stricto* and *Clostridium XI*, increases indole-3-acetic acid and indole, and induces AhR activation [152]. In the jejunum, tryptophan supplementation reduces the abundance of *Clostridium sensu stricto* and *Streptococcus* and increases the abundance of tryptophan metabolising *Lactobacillus* and *Clostridium XI*. This study also reported enhanced intestinal barrier function and the secretion of host defence peptides [153]. In agreement with these findings, [155] reported that increased dietary tryptophan is associated with increases in the expression of host defense peptides. Additionally, these authors observed an increase in α diversity indices, ACE and Chao1, and abundance of *Lactobacillus* in post-weaned pigs fed a diet containing 0.35% tryptophan compared to pigs fed diets containing 0.28, 0.21 or 0.14% tryptophan [155]. Furthermore, tryptophan supplementation to lipopolysaccharide (LPS)-challenged pigs exerts a range of beneficial effects, such as modulating the intestinal microbiota, improving villus height, villus area, barrier function and antioxidant capacity, activating the AhR pathway and also alleviating inflammation [163,164]. These studies highlight the potential beneficial effects of increased dietary tryptophan on microbiota composition, the production of AhR ligands, and on overall GIT health. Further research is warranted to evaluate the effects of tryptophan on the gut microbiota, the metabolites produced via microbiota tryptophan metabolism, and more precisely the exact effects of increased AhR activation. The modulation of the gut microbiota by amino acids is a relatively recent area of research and an area that requires further investigation. The effects of amino acid supplementation on the GIT microbiota in sows and pigs are presented in Table 6. Given the broad range of possible effects of amino acid supplementation, and the quantity of studies investigating their inclusion in sow and pig diets, only studies where the microbiota was analyzed have been included in Table 6.

#### 2.2.2. Nucleotides

Nucleotides are organic molecules that serve as precursors of DNA and RNA. Nucleotides have been recently suggested as an “overlooked prebiotic” that could potentially play a role in shaping the composition of the microbiota [37]. Interestingly, in vitro, nucleotides promote the growth and secretion of the biofilm of the probiotic *Lactobacillus casei*, while also enabling the crude extract of *Lactobacillus casei* to resist the biofilm formation of the pathogenic bacteria *Shigella* [37]. In mice, nucleotide supplementation promotes microbial diversity, while nucleotide-free diets enriched pathogenic bacteria, such as *Helicobacter*, and decreased beneficial bacteria, such as *Lactobacillus*, in feces [37]. In chickens, yeast nucleotides increase α diversity and the abundance of *lactobacillus* in the ileal microbiota [172]. Research investigating the effect of nucleotide supplementation on the pig’s microbiota is sparce. Nucleotides are present in the sow’s milk and may contribute to the establishment of the offspring’s microbiota [173,174]. Oral supplementation of nucleotides to pigs pre-weaning does not affect α diversity, but increases the fecal abundance of *Campylobacteraceae* and decreases *Streptococcaceae* at weaning [174]. However, the product utilized in this study, SwineMOD^®^ (Prosol, Madone, Italy), also contains yeast glucans which likely contribute to the effects on the microbiota [174]. Maternal nucleotide supplementation is associated with positive effects on offspring GIT health parameters, such as inflammation, intestine morphology and diarrhea occurrence [175]. However, the question of whether supplementing nucleotides in the maternal diet leads to alterations in the nucleotide composition of the milk and subsequently in the composition of the offspring’s microbiota remains to be answered.

Supplementing a pure nucleotide blend to 3-day-old weaned pigs results in dramatic changes in the colonic microbiota, reducing the *Firmicutes*: *Bacteroidetes* ratio and increasing the relative abundance of beneficial bacteria such as *Faecalibacterium*, *Blautia* and *Prevotella* [176]. Furthermore, the pure nucleotide blend increases the level of the SCFA acetic acid, isobutyric acid, isovaleric acid and valeric acid in the colon [176]. A nucleotide-rich yeast extract increases cecal *Lactobacillus* and colonic *Clostridium* cluster *IV*, and decreases cecal *Enterobacteriaceae* and colonic *Enterococcus* spp. when supplemented to pigs for the initial two weeks post-weaning [177]. However, the nucleotide-rich yeast extract product (Maxi-gen^®^, Canadian Bio-Systems, Canada) utilized in [177] contains a blend of yeast derivatives that may contribute to the modulation of the microbiota. The supplementation of a pure nucleotide blend to pigs weaned at 20 days has no effect on bacterial numbers in the jejunum, cecum or feces, although it does increase ADFI and plasma IgA [178]. Initial studies suggest that there is a potential role for dietary nucleotides in modulating the microbiota. However, further research utilizing pure nucleotides would be beneficial in order to advance the current understanding of their effects on the microbiota. The results from studies investigating the use of nucleotide-rich yeast blends, although displaying positive results, are difficult to interpret due to the lack of detail on the nucleotide composition of the product and the likely effects of alternative yeast derivatives present in the products. The effects of nucleotide supplementation on the GIT microbiota in the diet of pigs at different stages are presented in Table 7. Due to the broad scope of the modes of action for nucleotides, only studies where the microbiota was analyzed have been included Table 7.

#### 2.2.3. Polyphenols 

Polyphenols are secondary metabolites in plants and are particularly abundant in fruits, vegetables, grains and teas [179]. Polyphenols have established antioxidant and anti-inflammatory activities, as reviewed in [180]. Besides that, polyphenols have antimicrobial activity and can modulate the GIT microbiota when included in the diet of pigs [181]. As mentioned, polyphenols are deemed to have ‘prebiotic-like properties’ as they possess microbiota-modulating abilities when included in the diet. However, it is not clear if polyphenols are utilized directly by bacteria in the GIT, which is a requirement to be classed as a prebiotic, and so they are currently classed as “prebiotic-like”. Further research is required to investigate the mechanisms through which polyphenols modulate the GIT microbiota. The exact mechanism for the antimicrobial activity of polyphenols is unclear but, it likely occurs due to their interactions with the cell surface of the microbes [182]. In general, gram-positive bacteria are more sensitive to polyphenols than gram-negative bacteria [183,184]. 

Polyphenol supplementation has been associated with increases in the abundance of beneficial *Lactobacillus* [181,185], *Bifidobacteria* [185] and *Prevotella* [181] and decreases in abundance of harmful *Streptococcus* and *Clostridium* [186]. Feeding polyphenol-rich plant products to weaned pigs reduces the abundance of harmful bacteria, including *Streptococcus* and *Clostridium*, without affecting the abundance of the beneficial bacteria, *Lactobacillus* and *Bifidobacterium* [186]. However, supplementation can also lead to an increase in the pH of the feces and a reduction in the concentration of SCFA [186]. The decrease in SCFA noted may be a result of a decrease in *Bacteroidetes* abundance, which is a primary contributor to SCFA and promote a balanced microbiota, as polyphenol supplementation can decrease *bacteroidetes* in colonic digesta of weaned pigs [181]. The reduction in SCFA concentration and increase in the pH of the feces noted in [186] indicates a reduction in bacterial fermentation in the GIT. This is not a desirable effect as SCFA plays an essential role in the regulation of metabolism, the immune system, and cell proliferation in the GIT, while the increase in pH is not desirable as a lower pH in the intestine can help to limit the growth of pathogenic bacteria [187,188,189]. A combination of functional amino acids (arginine, leucine, valine, isoleucine, cysteine) with a polyphenol-rich extract from grape seed skins reduces microbial diversity. However, it increases *Lactobacillaceae* in the jejunum and SCFA production in the cecum, while reducing *Proteobacteria* in the cecum of pigs during the post-weaning phase [190]. Polyphenols have established microbiota modulation capabilities; however, results have been variable across different polyphenol types and sources. Further analysis is required to evaluate optimal sources and concentrations. Moreover, whether these effects on the microbiota are due to prebiotic mechanisms remains to be answered. Recent studies analyzing the effects of polyphenol supplementation in the diet of pigs at different stages on the GIT microbiota in pigs are presented in Table 8. Due to the broad scope of the modes of action displayed by polyphenols, only studies where the microbiota was analyzed have been included in Table 8.

## 3. Stimbiotics 

The concept of a stimbiotic was proposed in [23], where the authors suggested that certain bioactives, that were classed as prebiotics, may not be exerting their beneficial effects in the mode of action expected under the definition of a prebiotic [23]. Hence, they proposed a new class of bioactive which they termed “Stimbiotics”. When stimbiotics are included at low inclusion rates they promote increases in SCFA production disproportionally greater than if they were merely substrates for fermentation [23,194]. It is suggested that stimbiotics are pump primers, where they signal to fiber fermenting bacteria to increase their activity and thereby promote an increase in fiber fermentation. An example of a stimbiotic is XOS, which consists of chains of xylose linked by β(1–4) bonds [195]. The XOS is effective at modulating the GIT microbiota and improving performance when included in the diet of weaned pigs at inclusion rates as low as 0.02% [31]. Inclusion rates for NDO prebiotics, such as FOS, GOS and mannan oligosaccharide (MOS), can vary but are generally much higher than this, in the region of 0.1–0.2% [81,194]. Even at 10 or 20 times lower inclusion rates, stimbiotics can exert a greater effect on certain fiber fermentation parameters than certain prebiotics [194]. The use of XOS at a 0.007% and 0.01% inclusion rate has minor effects on the GIT microbiota and performance but overall results from trials suggest a higher inclusion rate of 0.02% or 0.04% to be more effective (Table 9) [31,196,197,198]. Stimbiotics are a relatively new concept and although a proportion of these bioactives have been studied as prebiotics, studies investigating their effect at the low inclusion levels associated with stimbiotic activity are limited, especially in the case of maternal supplementation where it is yet to be studied. Moreover, the low stimbiotic intake required to elicit changes in the microbiota makes them particularly interesting for inclusion in diets pre-weaning and immediately post-weaning, when intakes are generally low. XOS is currently the only recognized stimbiotic, further exploration is warranted to identify additional stimbiotics. The effects stimbiotic supplementation in the diet of pigs at different stages on the GIT microbiota and other health parameters are presented in Table 9.

## 4. Conclusions

The importance of the GIT microbiota is becoming increasingly evident, particularly with the strict new restrictions on antibiotic and antimicrobial use. Therefore, modulating the GIT microbiota through dietary intervention is a crucial area of exploration that can enhance animal health by increasing the production of host-health-promoting metabolites and limiting the proliferation of pathogenic bacteria. The benefits of prebiotic use illuminates their status as an intriguing bioactive group that can potentially act as alternatives to antibiotic and antimicrobial use on pig farms, particularly in the post-weaning phase. The benefit of prebiotics is evident. However, given the broad range of traditional prebiotics, combined with the growing list of newly classed novel prebiotics, the most effective prebiotics at the different stages of development need to be clarified. Particular attention should be placed on direct comparative research into different prebiotics and inclusion rates at critical periods of development. In addition, the mode of action of stimbiotics remains somewhat elusive. Given the potential for improved performance at such low inclusion rates, it is an area for increased exploration in the coming years. 

## Figures and Tables

**Table 1:** Effects of the inclusion of β-glucans in diets of sows and pigs at different stages of the life cycle on measures of GIT health and performance.

β-Glucan	Animal	Inclusion Rate *	Effect on Microbiota	Other Effects	Reference
Laminarin BG	Sows	1 g/day	Improved *Lactobacillus* spp. numbers in the offspring colon. Reduced offspring fecal counts of *Salmonella Typhimurium* post-challenge with *Salmonella Typhimurium*.	Improved offspring ADG and feed efficiency post-weaning. Improved offspring fecal scores. Increased total SCFA in offspring feces post-weaning.	[61]
Laminarin BG	Sows	1 g/day	Increased colonic *Lactobacillus* spp. gene numbers in offspring at weaning.	Increased villous height in offspring ileum D8 PW. Beneficial effect on immune gene expression markers in offspring pre-weaning and D8 PW. Increased offspring BW on D67 PW and improved G:F ratio from D16 to D117 PW.	[62]
Oat BG	Sows	150,000 g/ton	Significantly higher levels of *Bifidobacteria* in milk.	The *Bifidobacteria* isolate from the sow’s milk survived low pH and bile salts exposure when tested.	[63]
Yeast BG	Sows	1,000/2,000 g/ton	Not recorded.	Increased ADFI in both 1000 and 2000 g/ton BG groups compared to control. Increased ADFI in 1000 compared to 2000 g/ton. Weaning weight tended to increase in both groups. Inclusion rate of 1000 g/ton BG decreased TNF-α in sow and piglet serum compared to 2000 g/ton BG.	[64]
Yeast BG	Gilts	300 g/ton	Not recorded.	Increased IgA concentration and proliferation rate of intestinal epithelial cells from colostrum and milk. Did not modulate vaccine response. No effect on reproductive performance.	[65]
Oats BG	Suckling pigs	40 mg/kg bodyweight	No significant changes.	No effect on performance or gut morphology.	[66]
Algal BG	Weaned pigs	54/108 g/ton	Not recorded.	Inclusion rate of 108 g/ton reduced diarrhea frequency, decreased GIT permeability. Both inclusion rates enhanced the mRNA expression of tight junction proteins and boosted immune response against *Escherichia coli* infection.	[67]
Bacterial BG (High and low MW)	Weaned pigs	50 g/ton	Attenuated the impact of LPS infusion on total bacteria number and copy numbers of *Lactobacillus*, *Bifidobacterium*, *Bacillus* and *Escherichia coli* in the colonic digesta. Increased *Bifidobacterium* and *Bacillus* in the colonic digesta of LPS challenged pigs. High MW BG decreased *Escherichia coli* in colonic digesta of LPS challenged pigs.	Inhibited LPS-mediated depression in the growth performance, possibly via the Dectin-1 receptor and the TLR4/NF-κB pathway. Different effects were noted between high and low MW BG. Increased butyrate concentration.	[30]
Bacterial BG	Weaned pigs	500 g/ton	Increased abundance of *Lactobacillus* and *Bacillus* in the colon after ETEC challenge.	Increased TJP1 in jejunal epithelium following ETEC challenge. Decreased expression of inflammation related proteins in jejunal and ileal mucosa. Increased propanoic acid content in the colon.	[68]
Mushroom BG	Weaned pigs	Mushroom powder included at 2,000 g/ton (equivalent of 200 ppm BG)	No significant change.	Reduced feed intake.	[69]
Mushroom BG	Weaned pigs	Mushroom powder included at 2,000 g/ton (equivalent of 200 ppm BG)	Decreased relative abundance of *Prevotella*.	Improved gastrointestinal morphology and upregulated expression of nutrient transporters *SLC15A* and *FABP2* and tight junction protein *CLDN1*. Reduced ADFI no negative impact the ADG.	[33]
Rice Bran BG	Weaned pigs	1,000/2,000/4,000 g/ton	Linearly decreased coliform bacterial counts in feces with increasing BG inclusion.	Improved ADG and G:F ratio. Linear increase in nutrient digestibility from D0–D42 PW with increasing BG inclusion.	[70]
Yeast BG	Suckling/weaned pigs	Oral dose of 50–300 mg every two days. Dose rate started at 50 mg and increased weekly by 50 mg.	Modest effect on fecal microbiota with increases in *Methanobrevibacter*, *Fusobacterium* and a genus within the family of *Ruminococcaceae*. Difficult to state if positive or negative.	Did not affect vaccination response.	[71]
Yeast BG	Weaned pigs	250 g/ton	Increased abundance of pathogenic attaching and effacing *Escherichia coli* and decreased abundance of *Bifidobacterium* spp. in cecal digesta.	No effect on performance. Suppressed *IL10* expression in ileum.	[60]
Yeast BG	Weaned pigs	100 g/ton	Decreased fecal *Escherichia coli*.	Increased plasma leucocytes, increased lymphocyte proliferation and decreased TNF*-α* in the blood plasma at 2 and 4 h post LPS challenge.	[72]
Yeast BG	Weaned pigs	50 g/ton	Not recorded.	Attenuated the increase of IL-6 and TNF*-α* and enhanced IL-10 when pigs were challenged with LPS.	[50]
*Laminarin* *Hyperborean* BG	Grower pigs	250 g/ton	Reduced *Enterobacteriaceae* population in the ileum and colon.	Downregulated pro-inflammatory (*TNF-α*, *IL-1α*, and *IL-17A*) and anti-inflammatory (*IL-10*) markers in the colon.	[73]
*Laminarin digitata* BG	Grower pigs	250 g/ton	Reduced *Enterobacteriaceae* population in the ileum and colon.	Downregulated pro-inflammatory (*TNF-α, IL-1α*, and *IL-17A*) and anti-inflammatory (*IL-10*) markers in the colon. Reduced total volatile fatty acid concentration in ileum. Ex-vivo model showed an increase in *CXCL8* following LPS challenge.	
Yeast BG	Grower pigs	250 g/ton	Reduced *Enterobacteriaceae* population in the ileum and colon.	Downregulated pro-inflammatory (*TNF-α, IL-1α*, and *IL-17A*) and anti-inflammatory (*IL-10*) markers in the colon.	
Yeast BG	Grower pigs	500 g/ton	When challenged with *Salmonella enterica serovar Typhimurium*, the supplemented group had reduced shedding counts at D16 post-inoculation. Increase in several potential beneficial microorganisms in feces post-inoculation.	Not recorded.	[74]
Bacterial BG	Grower/finisher pigs	50/100/200 g/ton	Not recorded.	Inclusion rate of 100 g/ton improved ADG, FCR and nutrient digestibility. 100 and 200 g/ton inclusion rate increased carcass length. 100 g/ton improved pork quality.	[75]
Mushroom BG	Finisher pigs	Mushroom powder included at 1,000 g/ton (equivalent of 100 mg/kg BG)	Not recorded.	Reduced feed intake. Improved gain to feed ratio. Enhanced the color of fresh pork.	[76]

Updated from [77]. BG: beta glucan; ADG: average daily gain; SCFA: short-chain fatty acid; PW: post-weaning; BW: bodyweight; G:F gain: feed ratio; ADFI: average daily feed intake; α: alpha, IgA: immunoglobulin A; mRNA: messenger ribonucleic acid; MW: molecular weight; GIT: gastrointestinal tract; TNF: tumor necrosis factor; TJP1: tight junction protein 1; LPS: lipopolysaccharide; IL: interleukin; TLR: toll-like receptors; FCR: feed conversion ratio; ETEC: enterotoxigenic *Escherichia coli*. * Inclusion rate detailed in reference to complete feed, unless otherwise stated.

**Table 2:** Effects of NDO inclusion in the diets of sows and pigs at different stages of the life cycle on measures of GIT health and performance.

NDO	Animal	Inclusion Rate *	Effect on GIT Microbiota	Other Effects	Reference
Chito- oligosaccharides	Sows	30 g/ton	Not recorded.	Improved amino acid concentration in milk. Improved ADG and weaning weight of offspring. Reduced offspring hypoglycemia by stimulating hepatic gluconeogenesis.	[87]
Chito- oligosaccharides	Sows	30 g/ton	Not recorded.	Increased IgM in colostrum. Increased IgG and IL-10 in serum of offspring at weaning.	[88]
Fructo- oligosaccharide	Sows	1,500 g/ton	Not recorded.	Increased SCFA concentration in offspring feces during lactation and after weaning. Increased cecal goblet cell number and improved ileal cytokine secretions.	[89]
Galacto-oligosaccharide	Sows	10 g/day	Increased the abundance of *Alloprevotella* and *Ruminoclostridium_1* in sow feces and vertically increased fecal *Alloprevotella* and *Ruminoclostridium_1* in offspring feces.	Improved intestinal barriers, immune defense and ADG of offspring.	[90]
Mannan-oligosaccharide	Sows	400 g/ton	Not recorded.	Shortened wean to service interval. Improved growth performance and immunity in offspring.	[91]
Mannan-oligosaccharide	Sows	400 g/ton	No change.	Reduced inflammation marker expression and improved immune competence in offspring.	[92]
Chito- oligosaccharides	Weaned pigs	100/200/400 g/ton	All inclusion rates increased *Lactobacillus* counts in feces D14 and D21 PW. 200mg/kg COS decreased *Escherichia coli* counts in feces at D21 PW.	100 and 200 g/ton increased overall ADG, ADFI and G:F. All inclusion rates decreased incidence of diarrhea and diarrhea scores. 100 g/ton COS increased villus height in ileum and 200 g/ton COS increased villus height and villus height: crypt depth ratio in jejunum and ileum.	[93]
Chito- Oligosaccharides * Varying molecular weights	Weaned pigs	250 g/ton	The use of 5–10 and 10–50 kDa COS increased lactic acid bacteria populations in feces. Using 50–100 kDa COS decreased lactic acid bacteria populations in feces. Using 5–10, 10–50 and 50–100 kDa COS decreased *Escherichia coli* populations in feces.	Improved ADG and G:F D18-33 PW. Improved fecal scores D0-14 PW. Using 5–10 and 10–50 kDa COS increased nutrient digestibility of diets.	[94]
Chito- oligosaccharides	Weaned pigs	75/150/225 g/ton	Not recorded.	150 g/ton increased digestibility on D28 and D56 PW, increased villus height and villus height: crypt depth ratio on D28 and increased active cell division on D56 PW.	[95]
Fructo- oligosaccharide	Weaned pigs	40,000 g/ton	No change in fecal bacterial populations. Increase in fecal population of yeast.	Decreased fecal pH and increased organic acid concentration.	[96]
Fructo- oligosaccharide	Weaned pigs	2,500 g/ton	Reduced *Escherichia coli* and increased populations of *Bacillus* and *Bifidobacterium* in cecal digesta.	Improved ADG, apparent digestibility of crude protein, villus height in duodenum and expression of tight junction genes. Increased SCFA in cecal digesta. Decreased diarrhea incidence.	[97]
Galacto-oligosaccharide	Weaned pigs	500/1,000/1,500/2,000 g/ton	Increased the number of *Lactobacilli* and *Bifidobacteria* and decreased *Escherichia coli* in the feces in a linear or quadratic dose-dependent manner.	Improved growth performance in a linear or quadratic dose-dependent manner. Decreased serum pro-inflammatory cytokines in a quadratic dose-dependent manner and increased anti-inflammatory cytokines in a linear or quadratic dose-dependent manner. Promoted serum antioxidant activities in a linear or quadratic dose-dependent manner.	[81]
Lactulose	Weaned pigs	1,000/2,000 g/ton	Both inclusion rates increased *Lactobacillus* and reduced *Escherichia coli* fecal counts.	Both inclusion rates increased overall ADG and G:F but did not affect ADFI. Both inclusion rates improved nitrogen digestibility and gross energy.	[98]
Mannan-oligosaccharide	Weaned pigs	1,000 g/ton	Not recorded.	Improved growth and nutrient digestibility. Reduced diarrhea.	[99]
Xylo- oligosaccharide ^1^	Weaned pigs	10,000 g/ton	Increased the abundance of *Lactobacillus* and *Bifidobacterium* in the ileal digesta.	Reduced diarrhea. Increased ileal villus height and intestinal activity of antioxidizes. Reduced ileal and colonic content of IL-6 and increased colonic sIgA and IL-10 concentrations.	[100]

ADG: average daily gain; IgM: immunoglobulin M; IgG: immunoglobulin G; IL: interleukin; SCFA: short-chain fatty acid; COS: chito-oligosaccharides; ADFI: average daily feed intake; G:F gain to feed; PW: post-weaning; kDa: kilodalton; sIgA: secretory immunoglobulin A; XOS: xylo-oligosaccharide. ^1^ Higher inclusion rate means its mode of action could be both prebiotic and stimbiotic. * Inclusion rate detailed in reference to complete feed, unless otherwise stated.

**Table 3:** Effects of inulin inclusion in the diets of sows and pigs at different stages of the life cycle on measures of GIT health and performance.

Animal	Inulin Inclusion Rate *	Effect on GIT Microbiota	Other Effects	Reference
Sows	8,000/16,000/24,000 g/ton	Not recorded.	16,000 g/ton inclusion rate increased litter birth weight, reduced farrowing duration and reduced piglet deaths at birth. 8,000 and 16,000 g/ton inclusion rate increased litter weaning weight, piglet ADG and sow feed intake. Linear improvement in antioxidative status of the sow with increasing inclusion of inulin.	[109]
Sows	30,000 g/ton	Increased *Enterococci* in feces of sow and caecum of offspring. Deceased *Enterobacteria* and *Lactobacillus* amylovorus and increased *Eubacteria* and *Clostridium leptum* in offspring stomach digesta.	Decrease in sow fecal pH. Decreased concentrations of ammonia, n-butyric acid and i-valeric acid in the stomach digesta of offspring.	[114]
Weaned pigs	17,000 g/ton	Not recorded.	Improved daily gain and food efficiency D0–D7 PW.	[110]
Weaned/grower pigs	40,000 g/ton (Short-chain/long-chain/50:50 mixture of both)	All 3 types of inulin increased *Lactobacilli* and *Bifidobacteria* in the lumen contents in the distal colon. There was a strong effect of inulin on the abundance of *Lactobacilli* and *Bifidobacteria* in the mucosal microbiota. These mucosal microbiota alterations were evident as proximal as the jejunum in the short-chain inulin group. However, in the long-chain inulin group changes were not evident until the distal ileum or cecum.	All 3 types of inulin resulted in similar improvements. Improved iron utilization. Increased hemoglobin repletion efficiency. The cecum was the main site of inulin disappearance.	[111,112]
Grower pigs	30,000 g/ton	Did not alter numbers of *Lactobacilli, Bifidobacteria*, *Enterococci*, *Enterobacteria* or bacteria of the *Clostridium Coccoides*/*Eubacterium rectale group* in the duodenum, jejunum, or cecum. Reduced *Lactobacilli* in the stomach.	Reduced cecal acetate.	[113]
	50,000 g/ton	Increased β-diversity in colon and cecum. In total, 18 genera altered in the cecum, 17 in the colon and 6 in the ileum.	Increased SCFA in colon and caecum.	[115]
Finisher pigs	20,000 g/ton	Not recorded.	Improved antioxidant status and water holding capacity of meat, increased intramuscular fat.	[116]

ADG: average daily gain; PW: post-weaning; SCFA: short chain fatty acid. * Inclusion rate detailed in reference to complete feed, unless otherwise stated.

**Table 4:** Effects of resistant-starch inclusion in the diets of sows and pigs at different stages of the life cycle on measures of GIT health and performance.

Type of Resistant Starch	Animal	Inclusion Rate *	Effect on GIT Microbiota	Other Effects	Reference
RS1—High amylose corn	Sows	76.5% of total starch	Increased bacterial diversity in the sow feces.	Reduced birthweight but no difference in weaning weight. Increased serum triacylglycerol. Increased non-esterified fatty acids concentration and fat content in milk.	[123]
RS2—Field pea starch	Sows	33% of starch	Increased *Firmicutes*: *Bacteroidetes* ratio and abundance of *Bifidobacterium* in the sow feces. No microbiota differences in offspring.	Milk protein decreased and lactose increased in week 1 of lactation. Increased expression of *TJP1* in offspring. No other health benefits observed in offspring.	[124]
RS2—Field pea starch	Sows	33% of starch	No major differences could be distinguished.	Higher SCFA in colon of offspring.	[125]
RS2—Raw potato starch	Weaned pigs	5%	Increased *Clostridia* in feces.	Increased intestinal concentration of butyrate. Increased T-cell abundance and enhanced mucosal defense status in cecum.	[120]
RS2—Raw potato starch	Weaned pigs	0.5/1%	Not recorded.	Both inclusion rates improved fecal scores, however the 1% group had more solid feces D0–D14 PW. Both inclusion rates decreased ileal and cecal digesta pH.	[121]
RS2—Raw potato starch	Weaned pigs	7/14%	Both inclusion rates increased *Lactobacilli* and *Bacteroides* prevalence in the colon. No effect on colon lactic acid bacterial counts.	Increased ileum ammonia N concentrations. Resistant starch content of 7% and 14% improved fecal score D0–7 PW. Resistant starch content of 7% improved fecal score D0–D21 compared to control and 14% RS.	[122]
RS3—Retrograded tapioca starch	Grower pigs	Pregelatinized potato starch replaced by 34% retrograded tapioca starch	Not recorded.	Change in eating patterns but no increase in feed intake. Reduction in DE intake but no reduction in ADG.	[126]

SCFA: short-chain fatty acids; PW: post-weaning; DE: digestible energy; ADG: average daily gain. * Inclusion rate is detailed as described in publication. Inclusion rate detailed in reference to complete feed, unless otherwise stated.

**Table 5:** Effects of pectin inclusion in the diets and pigs at different stages of the life cycle on measures of GIT health and performance.

Pectin Source	Animal	Inclusion Rate *	Effect on GIT Microbiota	Other Effects	Reference
Source not stated (Yuzhung Biotech Corporation, China)	Weaned pigs (challenged with LPS)	50,000 g/ton	Improved α diversity and enriched anti-inflammatory and SCFA-producing bacterial groups in the ileal mucosa.	Ameliorated the LPS-induced inflammation response and damage to ileal morphology. Upregulated expression of *MUC2*. Increased acetate concentrations.	[128]
Citrus peel	Weaned pigs	50,000 g/ton	Increased *Lactococcus* and *Enterococcus* in the jejunum.	Improved intestinal integrity and reduced proinflammatory cytokines. Increased microbiota metabolites skatole, 3-indoleacetic acid, 3-indolepropionic acid, 5-hydroxyindole-3-acetic acid, and tryptamine. Metabolites activated the AhR pathway.	[129]
Apple	Weaned pigs	80,000 g/ton	Increased abundance of *Desulfovibrio* spp. and *Methanobrevibacter* spp. in the colon. The abundance of fungal keystone taxa with oxidative phosphorylation was decreased in the colon.	Decreased fecal redox potential. Increased the microbiota-derived antioxidant inosine.	[130]
Apple	Weaned pigs	11,800 g/ton	Not recorded.	Reduced gastric emptying and passage rate through the GIT. Increased digesta water content. Decreased retention time in small intestine. Increased SCFA content.	[131]
Apple	Grower pigs	50,000 g/ton	Reduced diversity at the genera level in the ileal mucosa. Increased abundance of potentially beneficial bacterial populations in the ileal and colonic mucosa. The alterations in the bacterial genera and fermentation metabolites were associated with the differentially expressed genes and cytokine in the ileum and cecum of pigs.	Reduced IL-6, IL-8, IL-12, and IL-18 and tended to reduce IFN-γ in the ileal mucosa. Reduced IL-1β and IFN-γ in the cecal mucosa and tended to reduce IL-8 and IL-1α. Reduced IL-6 in both the ileal and cecal mucosa. Upregulated *CLDN2*, tended to upregulate expression of *MUC2*, and downregulated *TLR2* and *NFKB* expression in the ileum. Increased *MUC2*, *TFF3*, *AMPK* and *TAK1* expression in the cecal mucosa. Increased sIgA content. Increased SCFA concentration in the cecum.	[132]

LPS: lipopolysaccharide; SCFA: short-chain fatty acid; AhR: aryl hydrocarbon receptor; GIT: gastrointestinal tract; IL: interleukin; sIgA: secretory immunoglobulin A. * Inclusion rate detailed in reference to complete feed, unless otherwise stated.

**Table 6:** Effects of amino acid supplementation to pigs at different stages of the life cycle on the GIT microbiota and measures of GIT health and performance.

Amino Acid	Animal	Inclusion Rate *	Effect on GIT Microbiota	Other Effects	Reference
Arginine	Sows	0.25%	Fecal α and β diversities remained the same. Increased both the *Bacteroidaceae* family and the *Bacteroides* genera in the feces.	Improved total number of pigs born. Tended to improve total born alive, reduce intrauterine growth restriction and mortality D0–D3.	[165]
Glutamate	Weaned pigs	0.5%	Increased relative composition of bacterial communities of the genus *Prevotella* and *Anaerovibrio* and decreased the genus *Clostridium* and *Terrisporobacter.*	Increased ADG, ADFI, and nutrient digestibility D0-D14 PW. Increased villus height: crypt depth ratio and number of goblet cells in the duodenum. Tended to increase villus height: crypt depth ratio and number of goblet cells in the ileum. Increased ileal gene expression of claudin family and Occludin. Decreased serum TNF-α and IL-6 and ileal gene expression of *TNF*.	[166]
Glycyl-glutamine	Weaned pigs	0.25%	Increased α diversity and abundance of beneficial anaerobes and fiber-degrading bacteria in the feces (stool from rectum).	Increased SCFA in the ileum and colon. Improved BW on D10 and D21 PW. Increased ADFI and ADG D0–D10 and D10–D21 PW. Reduced diarrhea ratio.	[167]
Tryptophan	Weaned pigs	0.2/0.4%	Increased α and β diversities and enriched abundances of *Prevotella*, *Roseburia* and *Sussinivibriogenera* in the cecum. Decreased *Clostridium sensustricto* and *Clostridium XI* in the caecum.	Inclusion rates of 0.2 and 0.4% increased the ADFI and ADG D0–D14 PW. Inclusion rates of 0.2 and 0.4% increased isobutyrate, isovalerate and indoleacetic acid in the colonic contents. An inclusion rate of 0.2% increased propionate in the colonic contents and indole in the cecal and colonic contents.	[152]
Tryptophan	Weaned pigs	0.1/0.2/0.4%	Inclusion rates of 0.2 and 0.4% enhanced Chao1 α diversity, reduced the abundances of *Clostridium sensustricto* and *Streptococcus* and increased the abundances of *Lactobacillus* and *Clostridium XI* in the jejunum.	Concentration of tryptophan in the serum increased in a dose dependent manner. Inclusion rates of 0.2 and 0.4% increased the abundances of ZO-1 and ZO-3, and the presence of claudin-1 proteins in the jejunum of weaned pigs was enhanced. An inclusion rate of 0.4% increased abundance of Occludin in the jejunum. An inclusion rate of 0.2% increased ZO-1 in the duodenum. An inclusion rate of 0.2% increased sIgA in the jejunum. Inclusion rates of 0.2 and 0.4% increased expression of porcine β-defensin genes in the jejunum.	[153]
Valine * In a low-protein diet	Weaned pigs	0.48% added to low-protein diet. (% SID valine: SID Lysine 0.12 above NRC recommended level)	Increased abundance *Mogibacterium* in colon.	Increased ADFI.	[146]
Valine + isoleucine * In a low-protein diet	Weaned pigs	0.48% valine and 0.33% isoleucine added. (% SID valine: SID lysine 0.12 above NRC recommended level, isoleucine equals NRC recommended level)	Increased abundance of *Actinobacteria, Enterococcus*, and *Brevibacillus* in colon.	Increased ADFI. Tended to increase final BW. Tended to increase ADG.	[146]
Citrulline	Finisher pigs	1%	Increased α diversity and microbiota composition of the feces. In particular, the altered gut microbiota at the phylum and genus level may be mainly involved in metabolic process of carbohydrate, energy, and amino acid, and exhibited a significant association with final weight, carcass weight and backfat thickness.	Drastically increased final BW, liveweight gain, carcass weight and average backfat. Decreased drip loss.	[168]
Glutamate ± arginine	Finisher pigs	1% glutamate ± 1% arginine	Glutamate in combination with arginine increased the abundance of *Actinobacteria* in the colon.	Glutamate alone or in combination with arginine decreased bodyfat weight and increased SCFA concentration in the colon.	[169]
Leucine	Finisher pigs	1% leucine	Increased the abundance of *Actinobacteria* in the colon.	Decreased body fat weight and increased colonic SCFA production.	[170]
Leucine and arginine		1% leucine + 1% arginine	Reduced abundance of *Bacteroidetes* and increased abundance of *Proteobacteria* in the colon. Increased *Clostridium_sensu_stricto*_1, *Terrisporobacter*, and *Escherichia-Shigella.*	Decreased body fat weight and increased colonic SCFA production.	
Leucine and glutamate		1% leucine + 1% glutamate	Increased propanoate concentration in colon.	No effect on performance or meat quality parameters.	

α: alpha; β: beta; ADG: average daily gain; ADFI: average daily feed intake; PW: post-weaning; TNF: tumor necrosis factor; IL: interleukin; SCFA: short-chain fatty acid; ZO: zonula occludens; sIgA: secretory immunoglobulin A; BW: bodyweight; SID: standardized ileal digestible. * Inclusion rate added to a basal diet formulated to meet requirements by recommended by NRC 2012, unless otherwise stated [171]. Inclusion rate detailed in reference to complete feed, unless otherwise stated.

**Table 7:** Effects of nucleotide inclusion in the diets and pigs at different stages of the life cycle on measures of GIT health and performance.

Nucleotide	Animal	Inclusion Rate *	Effect on Microbiota	Other Effects	Reference
Yeast nucleotides (SwineMOD^®^)	Suckling pigs	Oral dose of 100mg on D10, D15, D18 and D21 of life.	Increased abundance of *Campylobacteraceae*, and decreased abundance of *Streptococcaceae* in the feces at weaning but not at D12 PW.	A time- and tissue-dependent effect. Reduced inflammatory activation at weaning and increased erythropoietic activity post-weaning in blood transcriptome PW.	[174]
Equal ratios of CMP, UMP, AMP, GMP, IMP	Weaned pigs	350 g/ton	Reduced the *Firmicutes*/*Bacteroidetes* ratio in the colon. At genus level, enriched the relative abundance of *Prevotella*, *Faecalibacterium* and *Olsenella.*	Decrease the diarrhea rate. Increased villus height and the villus height: crypt depth ratio in the ileum. Upregulated protein expression of tight junction proteins and the mRNA expression of *MUC2* while the mRNA expression of *MUC4* was downregulated in the ileal mucosa. Increased the ileal mucosa genes expression of *IL21*, *INFG*, *IL10*, *IL4*, *IL6* and *TNF* and increased the protein expression of NF-κB, IL-6 and TNF-α. Increased short chain fatty acid in the colon.	[176]
Yeast nucleotides (Maxi-Gen^®^)	Weaned pigs	1000 g/ton	Clean room: suppressed growth of cecal *Enterobacteriaceae* members and colonic *Enterococcus* spp, improved proliferation of cecal *Lactobacillus* spp. and colonic *Clostridium cluster IV* and *XVIa* members. Unclean room: improved proliferation of cecal *Clostridium cluster IV* and suppressed proliferation of colonic *Enterococcus* spp.	No effect on growth performance in clean or unclean conditions. Clean room: tended to improve the villus height: crypt depth ratio. Unclean room: upregulation of ileal *PDCD1*, *IL1B*, *IL6*, *IL10* and *TNF*.	[177]
45.1 mg AMP, 22.4 mg CMP, 65.8 mg GMP, 9.5 mg IMP, and 1202.0 mg UMP	Weaned pigs	Oral dose of 1.34 g per day	Increased bacterial numbers of *Enterococcus* spp. in the cecal digesta. No difference in jejunum, cecum, or feces for numbers of total bacteria, *Lactobacillus* group, *Enterobacteriaceae*, *Bifidobacteria* spp., *Clostridium Cluster XIV*, or *Clostridium Cluster IV*.	Increased ADFI. Increased plasma IgA concentrations. No change in gut morphology.	[178]

PW: post-weaning; CMP: cytidine monophosphate; UMP: uridine monophosphate; AMP: adenosine monophosphate; GMP: guanosine monophosphate; IMP: inosine monophosphate; mRNA: messenger ribonucleic, IL: interleukin; TNF: tumor necrosis factor; acid ADFI: average daily feed intake; IgA: immunoglobulin A. * Inclusion rate detailed in reference to complete feed, unless otherwise stated.

**Table 8:** Effects of polyphenol inclusion in the diets and pigs at different stages of the life cycle on measures of GIT health and performance.

Polyphenol Source	Animal	Inclusion Rate *	Effect on Microbiota	Other Effects	Reference
Holly	Weaned pigs	250 g/ton	Increased the abundance of *Firmicutes* and reduced *Bacteroidetes* in the colon. Increased relative abundance of *Prevotella_9* in the caecum and *Lactobacillus* both in the caecum and colon of LPS challenged pigs.	Decreased villus height: crypt depth ratio. Increased jejunal lactase activity of LPS challenged pigs. Higher activity of sucrase and lactase in jejunum and sucrase in ileum. Reduced concentration of TNF-α, IL-6 and insulin in plasma. Increased glucagon concentration in plasma. Increased the mRNA expression of tight junction proteins. Increased the concentrations of cecal valerate and colonic acetate and isovalerate in LPS challenged piglets.	[181]
Citrus flavonoids	Weaned pigs	0.3 g/ton	Increased the abundance of several genera of bacteria such as *Lactobacillus*, *Roseburia*, and *Clostridium* in the cecum. Decreased the relative abundance of *Dorea*, *Desulfovibrio*, and *Actinobacillus*, among others, in the cecum.	Increased BW and ADG during the starter and entire period. Increased the expression of genes related to barrier function, digestive enzymes, and nutrient transport.	[191]
Grape pomace	Grower pigs	50,000 g/ton	Decreased *Lactobacillus* and *Ruminicoccus* and increased *Treponema* and *Campylobacter* in the colon.	Increased numbers of eosinophils induced by *Ascaris suum* infection in the duodenum, jejunum and ileum, and modulated gene expression in the jejunal mucosa of infected pigs.	[192]
Flavonoid enriched cocoa powder	Finisher pigs ^1^	0/2.5/10/20 g/d (flavanol concentration of 20.5 mg/g)	Rates of 10 and 20 g/d increased the abundance of *Lactobacillus* in the species and *Bifidobacterium* in the proximal colon.	No effect on bodyweights. Expression of *TNF* and *TLR2*, *TLR4*, and *TLR9* was reduced in the ileal Peyer’s patches, mesenteric lymph nodes and proximal colon.	[185]
Grape pomace (Anta^®^Ox E) or spent hops (Anta^®^Phyt H)	Weaned/ grower pigs	10,000 g/ton	Lower counts of *Streptococcus* spp. and *Clostridium Cluster XIVa* in the faecal microbiota.	Both showed an improved gain: feed ratio in comparison to the control group Both increased fecal pH value and lowered levels of volatile fatty acids.	[186]
Red-osier dogwood polyphenol	Finisher pigs	5,000 g/ton	Increased α-diversity, class *Bacilli*, order *Lactobacillales* and family *Lactobacillaceae* in ileal digesta. Within family *Lactobacillaceae*, *Lactobacillus* was the main responder by increasing from 5.92% to 35.09% in ileal digesta.	No effect on performance. Increased propionate in ileal digesta.	[193]

LPS: lipopolysaccharide; TNF: tumor necrosis factor; Il: interleukin; mRNA: messenger ribonucleic acid; BW: bodyweight; ADG: average daily gain. * Inclusion rate is detailed as described in publication. Inclusion rate detailed in reference to complete feed, unless otherwise stated. ^1^ 5-month-old pigs, 28 kg mean bodyweight.

**Table 9:** Effects of stimbiotic inclusion in the diets of sows and pigs at different stages of the life cycle on measures of GIT health and performance.

Stimbiotic	Inclusion Rate *	Animal	Effect on GIT Microbiota	Other Effects	Reference
Xylo- oligosaccharide	100 g/ton	Weaned pigs	Enhanced α-diversity, reduced *Lactobacillus,* and increased *Streptococcus* and *Turicibacter* at the genus level in the distal gut digesta.	Reduction in the inflammatory marker IFN-γ and improved intestinal barrier function through up regulation of *TJP1*. Little effect on growth performance, intestinal morphology, blood cells and biochemical markers.	[197]
Xylo- oligosaccharide	200 g/ton	Weaned pigs	Decreased *Escherichia coli* and increased *Lactobacilli* fecal shedding on D14 PW but not on D28 PW.	Improved ADG, FCR and digestibility. Increased villus height: crypt depth ratio in jejunum.	[31]
Xylo- oligosaccharide	400 g/ton	Weaned pigs	Not recorded.	Improved ADG and weight D28 PW. Increased serum glucose content. Decreased blood urea nitrogen and triglyceride. Increased serum IgG. Improved antioxidant and immune function of pigs.	[198]
Xylo- Oligosaccharide + β-1,4-endo xylanase (VistaPros^®^)	100 g/ton	Weaned/grower pigs	Increased relative abundance of beneficial bacteria *norank_f_Muribaculaceae*, *Rikenellaceae_RC9_gut_group*, *Parabacteroides*, and *unclassified_f__Oscillospiraceae* in the feces on D42 PW.	Improved BW of piglets on D28 and D42 PW and increased ADG and ADFI from D14–28 PW and from D0–D42 PW. Increased plasma insulin-like growth factor on D42 PW.	[199]
Xylo- Oligosaccharide + β-1,4-endo xylanase (Signis^®^)	100 g/ton	Weaned/grower pigs	Increased proportion of *Clostridiales Family XIII Incertae Sedis* and *Clostridiaceae* (Families contain beneficial fibrolytic and butyrate-producing bacteria) in the feces on D35 PW.	Increased weight at D42 PW under poor sanitary conditions (prebiotic comparisons included at 10- and 20-times higher doses did not increase bodyweight). Increased VFA:BCFA higher than prebiotics. Reduced plasma TNF-α content in pigs raised under poor sanitary conditions.	[194]
Xylo- Oligosaccharide (XOS) + β-1,4-endo xylanase (XYL)	XYL: 150 g/ton XOS: 200 g/ton XYL + XOS: 150 g/ton + 200 g/ton	Grower pigs	Limited effects. No effect on α or β diversity. Operational taxonomic units associated with *Muribaculaceae_ge* and *Prevotellaceae NK3B31* group were higher in the feces of pigs from all 3 diets.	XYL: no effect on performance.. XOS: limited effect on growth performance. Reduced G:F D0-D7 of trial. Improved ADG D7-D14 of trial. No overall affect (D0-D35). XYL + XOS: no interaction.	[196]
Xylo- oligosaccharide	100/250/500 ^1^ g/ton	Grower/finisher pigs	100 g/ton decreased presumed pathogenic bacteria, *Proteobacteria* and *Citrobacter*, and increased likely beneficial bacteria, *Firmicutes* and *Lactobacillus*, abundance in the colonic contents of pigs in the grower to finisher phase (30–100 kg). The dose and exposure time to XOS affected colonic microbial communities.	100 g/ton increased acetic acid and total SCFA concentration in the intestinal contents.	[200]

ADG: average daily gain, SCFA: short chain fatty acid, FCR: feed conversion ratio, PW: post-weaning, IgG: immunoglobulin G, BW: bodyweight, ADFI: average daily feed intake, VFA: volatile fatty acids, BCFA: branched chain fatty acids, G:F: gain to feed ratio, TNF: Tumor necrosis factor. * Inclusion rate is detailed as described in publication. Inclusion rate detailed in reference to complete feed, unless otherwise stated. ^1^ Higher inclusion rate means its mode of action could be both prebiotic and stimbiotic.

## Data Availability

Not applicable.
